# Pathogenicity of the Fungus, *Aspergillus clavatus*, Isolated from the Locust, *Oedaleus senegalensis*, Against Larvae of the Mosquitoes *Aedes aegypti, Anopheles gambiae* and *Culex quinquefasciatus*

**DOI:** 10.1673/031.009.5301

**Published:** 2009-07-13

**Authors:** Fawrou Seye, Oumar Faye, Mady Ndiaye, Ebrima Njie, José Marie Afoutou

**Affiliations:** ^1^ U.E.R.B.V., Laboratory of Reproduction Biology, Animal Biology Department, Faculty of Sciences and Technics, Cheikh Anta Diop University of Dakar, Senegal. P.O. Box: 5005, Dakar Fann, Senegal; ^2^ Laboratoire d'Histologie, Embryologie et Cytogénétique, Faculté de Médecine, Pharmacie et d'Odontostomatologie, Université Cheikh Anta Diop de Dakar; ^3^ Department of Agriculture and Biological Sciences P.O. Box: 3530 Gambia, University of the Gambia

**Keywords:** biological control, entomopathogenic fungi

## Abstract

The use of insect pathogenic fungi is a promising alternative to chemical control against mosquitoes. Among the Hyphomycetes isolated from insects for mosquito control, the genus *Aspergillus* remains the least studied. In September 2005, four fungi were isolated from the Senegalese locust, *Oedaleus senegalensis* Kraus (Orthoptera: Acrididae), collected in Dakar, Senegal. One of these fungi, identified as *Aspergillus clavatus*, Desmazières (Eurotiales: Trichocomaceae) was highly pathogenic against larvae of the mosquitoes *Aedes aegypti* L., *Anopheles gambiae* s.l. Giles and *Culex quinquefasciatus* Say (Diptera: Culicidae). An application of 1.2 mg/ml dry conidia yielded 100% mortality after 24 hours against both *Ae. aegypti* and *Cx. quinquefasciatus* while with *An. gambiae* it was 95%. With unidentified species in the genus *Aspergillus*, mortality after 24 h was <5% against all the larval species. Application of *A. clavatus* produced in a wheat powder medium using doses ranging between 4.3 to 21×107 spores/ml, caused 11 to 68% mortality against *Cx. quinquefasciatus* at 24h, and 37 to 100% against *Ae. aegypti*. Microscopic observations showed fungal germination on both *Ae. aegypti* and *Cx. quinquefasciatus* larvae. Histological studies revealed that *A. clavatus* penetrated the cuticle, invaded the gut and disintegrated its cells. Some *Cx. quinquefasciatus* larvae, treated with *A. clavatus* reached the pupal stage and produced infected adults. However, the infection was mainly located on the extremity of their abdomen. These results suggest that *A. clavatus* could be an effective tool to manage mosquito proliferation.

## Introduction

Mosquito-borne diseases currently represent a great health threat in tropical and subtropical climates. As an alternative to chemical insecticides, natural products (Koua, 1998; Batra et al., 1998; Ravindra et al., 2002), predatory fish (Legner 1995; Karch and Coz 1983; Fillinger et al. 2003) and entomopathogenic fungi (Nnakumusana 1985; Su et al. 2001; Scholte et al. 2003) are frequently used in mosquito control. Fungi such as *Metarhizium anisopliae* (Metchnikoff) Sorokin (Moniliales) and *Beauveria bassiana* (Balsamo) Vuillemin (Clavicipitaceae), commonly found on terrestrial insects can also kill mosquito larvae (Alves et al. 2002; Silva et al. 2004a). Studies have shown that mosquito larvae are susceptible to infections by fungi such as *Leptolegna chapmanii* (Lord and Fukuda, 1988), *M. anisopliae* (Riba et al. 1986; Lacey et al. 1988; Alves et al. 2002; Silva et al. 2004a; Wright et al. 2005), *B. bassiana* (Alves et al. 2002), *Aspergillus parasiticus* (Hati and Ghosh 1965), *Aspergillus* spp. (de Moraes et al. 2001), *Aspergillus flavus, A. parasiticus, Penicillium falicum, vasinfectum, Fusarium vasinfectum* and *Trichoderma viride* (Govindarajan et al. 2005). Among the various *Aspergillus* species known to infect mosquitoes, *A. clavatus* Desmazières (Eurotiales: Trichocomaceae) has not been examined as a possible biological control agent. This study assessed the pathogenicity of an *A. clavatus* strain against larval stages of various species of mosquitoes.

## Materials and Methods

### Isolation

In September 2005, locusts of the species *Oedaleus senegalensis* Kraus (Orthoptera: Acrididae) were collected from plants growing near the department of Animal Biology (University CHEIKH A. DIOP, Dakar- Senegal). They were killed and placed for 24 hours on soil collected from the botanical garden to allow saprophytic fungi attack. Afterward, they were placed in Petri dishes containing 20 mg of wheat flour (locally purchased and sterilized in the autoclave for about 15 min at 120°C) mixed with 15 ml of sterile distilled water. One Petri dish containing the same medium only was used as a control. They were maintained at 26°C average temperature and in the range of 80– 85% ambient RH. Four days later, 4 fungal isolates appeared in the plates containing the insects, but not on the control. These fungi were separately cultivated in Petri dishes on the same medium. Dry conidia were harvested from the surface of the medium directly by scraping and conserved in Pyrex bottles sterilized at 120°C.

Fungi were identified according to Rapper and Fennel (1965), Samson (1979) and Guarro et al. (1999). The fungus *A. clavatus* was easily identified by his long phototrophic conidiophores (Yaguchi et al., 1993) on microscopic examination during germination.

### Preliminary fungal tests on the mosquito larvae (Bioassay 1)

In September 2005, larvae of *Aedes aegypti* L., *Anopheles gambiae* s.l. Giles and *Culex quinquefasciatus* Say (Diptera: Culicidae) were collected from various vats containing rainwater. For each fungal isolate, 1.2 mg/ml dry conidia were applied to 25 larvae (3^rd^ and 4^th^ instar) in 9 × 1.5 cm Petri dishes sterilized at 120°C and containing sterile 25 ml of tap water. There were four replicates for each treatment. Four non-treated Petri dishes served as control. The more virulent *A. clavatus* fungus was selected for production and application.

### Microscopic observations

Larvae of *Ae. aegypti* and *An. gambiae* treated with *A. clavatus* were fixed after dying, sectioned, mounted, and then observed under light microscopy. The larvae of *Cx. quinquefasciatus* treated with *A. clavatus* at 1.2 mg/ml were used for cuticle observations under light microscopy. Larvae treated with three drops of the aqueous spore solution (1.2 mg/ml), were incubated on wheat powder medium (for *Ae. aegypti*) and on wet filter paper (for *Cx. quinquefasciatus*) at 85% R.H. and 26°C followed by observation of fungal germination.

### Fungal production

*A. clavatus* was grown in Petri dishes containing 20 g of wheat powder (sterilized for 15 min at 120°C) and 15 ml of sterile distilled water. After four days incubation at 26°C, the substrate and dry conidia content were mixed to obtain a powder (conidia - wheat powder mixture). The number of conidia was determined using a haemocytometer.

### Application of fungus (Bioassay 2)

*Ae. aegypti* and *Cx. quinquefasciatus* mosquito larvae were collected from vats containing rainwater in late November 2005. *An. gambiae* larvae were scarce at this time and were not used for the bioassay. Six plastic bottles (10 × 10 × 7 cm), each containing 500 ml of sterile tap water and 50 larvae (L3 and L4), were used for each mosquito species. Larvae were then treated with *A. clavatus* conidial mixture at 4, 8, 12, 16 and 20 g/l. The corresponding concentrations were 4.3; 8.5; 13; 17 and 21 × 10^7^ spores/ ml respectively. Culture medium (wheat powder only) at 0.02 mg/ml served as control for each treatment. Total mortality was recorded for all replicates of each treatment at 24 hours post-inoculation. The surviving larvae were reared in plastic boxes (10 × 10 × 7 cm) containing 500 ml of tap water for 7 days. The emerging adults were incubated for 24 h at 26°C and fungal germination was observed after microscopic examination.

### Data analysis

Data on mortality were corrected with Abbott's formula (Abbott, 1925). Student's t-test was used to compare mortality for *Ae. aegypti* and *Cx. quinquefasciatus*.

**Figure 1.**
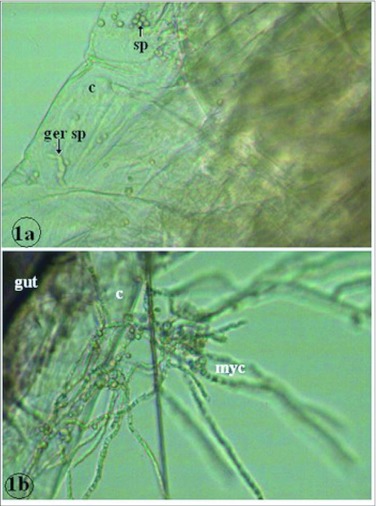
Larvae of *Culex quinquefasciatus* infected by *Aspergillus clavatus* via cuticle (la and l b). ×400. c = cuticle, ger sp = germinating spore, myc = mycelium, sp = spore.

## Results

Four *Aspergillus* species were isolated from the dead locusts. Preliminary tests (Bioassay 1) with conidia suspensions of each isolate revealed that *A. clavatus* was highly pathogenic against larvae of *Ae. aegypti, Cx. quinquefasciatus* and *An. gambiae*. Mortality rates were 100% against both *Ae. aegypti* and *Cx. quinquefasciatus*, while against *An. gambiae* it was 95%. All rates were in comparison to the control mortality (< 5%) after 24 hours (Table 1). With the other isolates (S1, S2 and S4), also identified as species in the genus *Aspergillus*, infection against the larvae was less than 5%.

*A. clavatus* induced significant mortality against mosquito larvae (Table 2) when applied (Bioassay 2 in relative humidity ranging from 65 to 80%, and temperature ranging from 24 to 26°C;. The mortalityt varied from 10.6 to 68% for *Cx. quinquefasciatus* and 36.7 to 100% for *Ae. aegypti*. Larval mortality was significantly higher against *Ae. aegypti* than *Cx. quinquefasciatus* (P = 0.0001) (Table 2). *A. clavatus* infection was observed under the microscope. On dead larvae, spores were found attached to the cuticle of *Cx. quinquefasciatus* (Figure 1a). Germinating spores (Figure 1a) and mycelia (Figure 1b) were found growing on *C quinquefasciatus* larva.

**Table 1.**
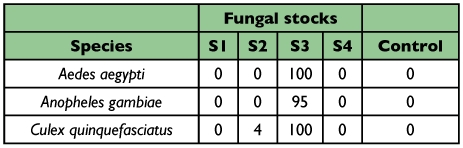
Larval mortality 24 h after fungal test on the mosquito larvae Aedes aegypti, Anopheles gambiae and Culex quinquefasciatus (average of four replicates).

**Figure 2.**
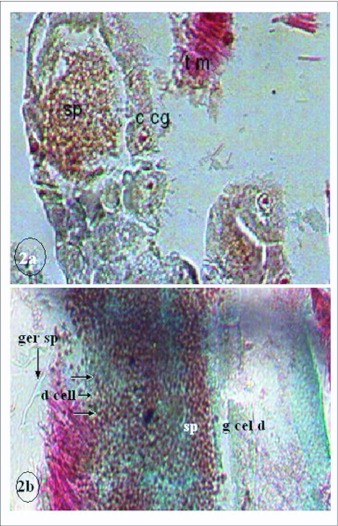
*Anopheles gambiae* gastric cacae (2a) and Aedes *aegypti* gut (2b) after infection by *Aspergillus clavatus*. × 400. c cg = cell of gastric caecum, d cell = disintegrating cells, g cel d= gut cell in disintegration, t m= tissue of muscle.

Histological studies revealed that the gastric caeca of some *An. gambiae* was invaded by *A. clavatus* spores (Figure 2a). Gut invasion by conidia and initial stages of germinating conidia were observed on *Ae. aegypti* larvae (Figure 2d). The fungus penetrated the cuticle of *Ae. aegypti* larvae. Conidial germination was also observed on *Cx. quinquefasciatus* larvae incubated on wet filter paper and larvae of *Ae. aegypti* incubated on sterile wheat flour. Fungal growth was observed on all treated and incubated larvae.

Occasionally, a low percentage of *Cx. quinquefasciatus* larvae treated with *A. clavatus* conidia were able to pupate and produce adults. Germinating conidia was observed on the tip of adult abdomen 24h after incubation.

## Discussion

*A. clavatus* was more virulent to the mosquito larvae than the other three fungal isolates. Laboratory results showed that, *A. clavatus* was highly pathogenic against larvae of *Ae. aegypti, An. gambiae* and *Cx. quinquefasciatus*. However, these mosquito larvae do not have the same susceptibility to the fungus. With the same dry conidial dose, (1.2 mg/ ml), death rate was 100% against both *Ae. aegypti* and *Cx. quinquefasciatus* larvae and 95% against *A. gambiae* larvae. Referring to larval species susceptibility, the effect of this fungus is similar to that of *M. anisopliae* (Moniliales) and *Tolypocladium cylindrosporum* (Hypocreales) against *An. Stephensi, Cx. pipiens* and *Ae. aegypti* larvae (Riba et al., 1986).

**Table 2.**
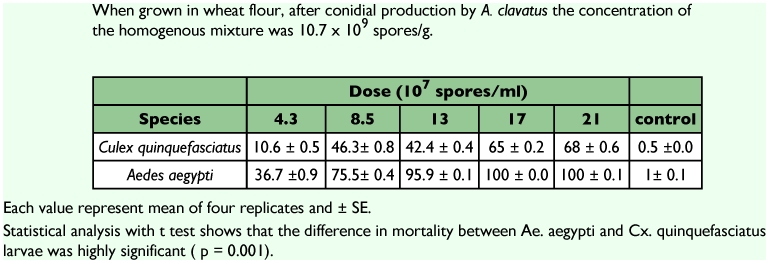
Percent mortality rates for Culex quinquefasciatus and Aedes aegypti larvae treated with mixture of Aspergillus clavatus (average of four replicates).

When treated with *A. clavatus* spores, mortality was 68 % against *Cx. quinquefasciatus* and 100% against *Ae. aegypti* for 21 × 10^7^ spores/ml. Bisht et al. (1996) found that the fungus *Leptolenia caudata* (Oomycetes) yielded a LD_100_Of 7.10^3^ spores/ml against *An. culicifacks* after 7 days. Riba et al. (1986) obtained a LD_100_ in the order of 10^7^ spores/ ml with a stock of *M. anisopliae* against *Ae. aegypti* larvae within 26 hours. From our results the LD_100_ against *Ae. aegypti* was closer to that of *M. anisopliae* against *Ae. aegypti*. Observations of larvae treated with *A. clavatus* revealed that just after adhesion of conidia on the cuticle, some germinated. The conidial proliferation on the cuticle became more obvious after 48 hours that is similar to previous studies on insects (Brett *et al*. 2004). Silva et al. (2004a) showed that larvae of mosquito treated with *M. anisopliae* had high amounts of conidia adhering to the colloid chitin with at least 90 % germination after 24 hrs incubation.

High levels of germination occurred on *A. clavatus* on dead *Ae. aegypti* larvae incubated on medium for 48 to 72 hours. Fungal germination was also observed on *Cx. quinquefasciatus* larvae in contact with aqueous solution of *A. clavatus* spores and incubated on wet filter paper, which is in agreement with Silva et al. (2004b).

However, the cuticle does not represent the only way for fungal infection. Other possible routes of invasion for *M. anisopliae* have been identified in mosquitoes via the respiratory siphon or the alimentary canal (Al-Aidroos and Roberts, 1978; Lacey et al., 1988; Silva et al., 2004b). Our histological results revealed a high gastric caeca invasion by *A. clavatus* spores in *An. gambiae* larvae. In the digestive tract for *Ae. aegypti* larvae, it was observed germinating conidia, rupture and disintegrating cells of gut. This has also been reported in previous histological studies (Lacey et al. 1988; Lord and Fukuda 1988; Silva et al. 2004b). According to Crisan (1971) and Lacey et al. (1988), a partial digestion of fungal conidia in the gut may induce a release of toxic substances. Silva et al. (2004b) revealed that rupture and disintegration of cells in the gut on dead larva might be due to the chitinolytic enzymes or others substances produced by the spores. According to Hajek and St. Leger (1994), aggressiveness of entomopathogenic fungus is related to proteolytic, lipolytic and chitinolytic mechanisms that can act after conidial adhesion on the larval cuticle or after invasion of the gut (Crisan 1971; Lacey et al. 1988; Domnas and Warner 1991; Silva et al. 2004b). *A. clavatus* produces a number of secondary metabolites as tryptoquivaline and tryptoquivalone (Clardy et al. 1975; Buchi et al. 1977); cytochalasin (Demain et al. 1976; Steyn et al. 1982; Lopez-Diaz and Flannigan 1997) and patulin (Varga et al. 2003). The pathological effects noted on the larvae treated with *A. clavatus* in our experiments might be due to these substances.

Light microscopy observations showed that *A. clavatus* conidia produce germ tubes on *Cx. quinquefasciatus* larval cuticle and germinate. Sweeney (1978) showed that with temperatures higher than 3O°C, spores of *Culicinomyces* sp. could adhere to the cuticle and invade the gut of *An. amictus* or that of *Cx. fatigans* larvae. This would explain the speed of *A. clavatus* germination on larvae incubated at temperatures ranging between 24 and 26°C.

*Cx. quinquefasciatus* larvae treated with *A. clavatus* could pupate and produce adults. The resulting adults were collected seven days later and incubated and displayed fungal germination on their abdominal extremities There was no fungal germination on adults that resulted from untreated larvae (control). This suggests that adult mosquitoes that result from treated larvae are likely contaminated at a pre-imaginal stage. Such an observation was also reported in previous studies on adult mosquitoes. Indeed *Ae. albopictus* larvae (Laird et al. 1992) and *Ae. aegypti* larvae in contact with fungus such as *Coelomomyces* could pupate and produce infected adults (Lucarotti and Shoulkamy 2000). According to Laird et al. (1992), infection of adult *Ae. albopictus* by the fungus *Coelomomyces stegomyiae var stegomyiae* could be mortal.

However, in our study, no mortality was recorded for adults reared during 7 days. Lucarotti (1992) found that, on adult mosquitoes, infection by *C. stegomyiae* targets mainly the ovaries, which may explain the germination of *A. clavatus* on the tip of the abdomen.

The laboratory results show that *A. clavatus* isolated from *O. senegalensis*, is virulent against *Ae. aegypti, An. gambiae* and *Cx. quinquefasciatus* larvae and could be developed as a biological control agent against mosquitoes. However, further studies are needed for *A. clavatus* strain optimization, and development of better substrates for mass production and practical use. Characterization and application of toxins on mosquito larvae are needed to better understand their rapid killing effects.
